# Analysis and Optimization for Downlink Cell-Free Massive MIMO System with Mixed DACs

**DOI:** 10.3390/s21082624

**Published:** 2021-04-08

**Authors:** Meng Zhou, Yao Zhang, Xu Qiao, Weiqiang Tan, Longxiang Yang

**Affiliations:** 1 The Department of Wireless Communication Key Lab of Jiangsu Province, Nanjing University of Posts and Telecommunications, Nanjing 210003, China; 2018010202@njupt.edu.cn (M.Z.); 2017010201@njupt.edu.cn (Y.Z.); 1017010132@njupt.edu.cn (X.Q.); 2 School of Computer Science and Cyber Engineering, Guangzhou University, Guangzhou 510006, China; wqtan@gzhu.edu.cn

**Keywords:** AQNM, cell-free massive MIMO, conjugate beamforming, mixed DACs, weighted max-min scheme

## Abstract

This paper concentrates on the rate analysis and optimization for a downlink cell-free massive multi-input multi-output (MIMO) system with mixed digital-to-analog converters (DACs), where some of the access points (APs) use perfect-resolution DACs, while the others exploit low-resolution DACs to reduce hardware cost and power consumption. By using the additive quantization noise model (AQNM) and conjugate beamforming receiver, a tight closed-form rate expression is derived based on the standard minimum mean square error (MMSE) channel estimate technique. With the derived result, the effects of the number of APs, the downlink transmitted power, the number of DAC bits, and the proportion of the perfect DACs in the mixed-DAC architecture are conducted. We find that the achievable sum rate can be improved by increasing the proportion of the perfect DACs and deploying more APs. Besides, when the DAC resolution arrives at 5-bit, the system performance will invariably approach the case of perfect DACs, which indicates that we can use 5-bit DACs to substitute the perfect DACs. Thus, it can greatly reduce system hardware cost and power consumption. Finally, the weighted max–min power allocation scheme is proposed to guarantee that the users with high priority have a higher rate, while the others are served with the same rate. The simulation results prove the proposed scheme can be effectively solved by the bisection algorithm.

## 1. Introduction

In recent years, cell-free massive multi-input multi-output (MIMO) has been considered as one of the most potential and disruptive technologies for the future beyond fifth-generation (B5G) and sixth-generation (6G) wireless communications [1,2,3], since it can significantly satisfy the ever-increasing requirements for high spectral efficiency (SE), coverage probability, and energy efficiency (EE) [4,5,6].

Different from the traditional massive MIMO, cell-free massive MIMO allows all access points (APs) to coherently and jointly serve a small number of distributed users in the same time-spectrum resource blocks. It can inherit the vital properties of collocated and distributed massive MIMO, such as channel hardening, enormous macro-diversity, and favorable propagation [5,6,7]. Therefore, this seminal technology received special attention from both the industry and academia in recent years. Assuming all APs used simple conjugate beamforming on the downlink and matched filtering on the uplink, the cell-free massive MIMO was first proposed in [3]. Based on the derived rate expression, a max–min power allocation scheme was applied to ensure that geometrically different users uniformly enjoy good quality of service (QoS). In order to maximize the minimum user rate for the filter-aided uplink cell-free massive MIMO systems, the above-mentioned scheme was also investigated in [8], where it can be decoupled into two sub-problems, i.e., receiver filter coefficient design and effective power allocation. The simulated results demonstrated that the proposed method can significantly improve both the fairness between different users and the sum rate. Because the multigroup multicast can provide an effective solution to transmit data to many groups of users at the same time, a multigroup multicast cell-free massive MIMO was investigated with distributed conjugate beamforming and short-term power constraint (STPC) [9]. It was shown that the STPC outperformed the long-term power constraint when the number of groups was small. Based on [9], a max–min power control algorithm was considered in the downlink multigroup multicast cell-free massive MIMO [10]. Apart from the above works that used traditional orthogonal multiple access (OMA), the non-orthogonal multiple access (NOMA) was also explored in cell-free massive MIMO, since multiple users can be severed in the same frequency/time/spatial resource block [11,12,13,14]. The achievable rate and the probability of successful successive interference cancellation (SIC) were obtained by employing the stochastic geometry approach [13]. In addition, the work [14] investigated the SE maximization problem for NOMA-aided cell-free massive MIMO, and a sequential convex approximation (SCA) method was developed. In addition, in [15], a downlink cell-free massive MIMO system with conjugate beamforming was investigated. With the effects of backhaul power consumption and the number of antennas at each AP, it investigated the total EE maximization problem. Furthermore, the derived results showed that the proposed algorithm can double the total EE compared with the equal power allocation. Different from [15,16], the works of [17,18] considered the zero-forcing precoding schemes, and the work in [19] investigated the millimeter-wave band.

All the above works showed that cell-free massive MIMO has remarkable advantages in both SE and EE, and they all advocated ideal hardware. In other words, all these works assumed that the perfect analog-to-digital converters (ADCs) or perfect digital-to-analog converters (DACs) were used. However, due to the fact that the hardware cost and circuit power consumption linearly increase with the sampling rate, and exponentially with the number of quantization bits of the ADCs/DACs [20,21,22,23], they will become a bottleneck for the future cell-free massive MIMO systems. To resolve this issue effectively, several novel architectures, such as low-resolution ADCs/DACs and hybrid precoding aided cell-free massive MIMO were proposed, and much effort was made to understand the behaviors of low-resolution ADCs/DACs [24,25,26,27,28]. Leveraging on the additive quantization noise model (AQNM), in [25], the authors took the first attempt to introduce low-resolution ADCs in cell-free massive MIMO systems and derived the achievable rate expression. To maximize the sum-rate expression, a max–min fairness power algorithm with ADC resolution allocation was proposed. Different from [25], the Bussgang decomposition theorem was introduced in [27,28]. In reality, the AQNM was more convenient for multi-bits quantization than the Bussgang.

Although a consensus was reached that low-resolution ADCs/DACs can effectively reduce energy consumption and hardware cost, 1-bit quantization will lead to significant performance loss in SE, especially in the high signal-to-interference- noise ratio (SINR) regime [22]. In addition, a pure low-resolution DAC architecture will also inevitably cause several other problems, such as error floor for linear multi-user detection, phase/frequency synchronization, and complex channel estimations [29]. The mixed-DAC architectures, in turn, were put forward in [30,31]. Although the mixed DAC was studied widely for traditional massive MIMO systems in the past few years, as far as the authors are concerned, the mixed-DAC architecture cell-free massive MIMO systems have not been given enough attention, and rarely do works explore this regard. Inspired by the above literature review, in this work, a downlink cell-free massive MIMO system with mixed DACs is considered.

Particularly, the main contributions of this paper are stated as:

(1) With the conjugate beamforming and AQNM, the tight closed-form downlink rate expression is derived based on the minimum mean square error (MMSE) channel estimation technique. It reflects the effect of the APs’ number, the downlink transmitted power, the number of quantization bits, and the proportion of perfect DACs in the mixed-DAC architecture;
(2) Considering the fact that, in the future, emerging situations such as telemedicine, driverless, and real-time operations, these situations often require high priority, while the remaining are served at the same rate. Then, the weighted max–min power allocation scheme is proposed. As the objective function of the optimization problem can be effectively proved to be quasi-concave, then the critical bisection algorithm is used to solve it;
(3) Numerical results are given to verify the derived results and prove that the proposed scheme can be effectively solved by the bisection algorithm.

The remainder of this paper is organized as follows. Section 2 introduces the considered downlink cell-free massive MIMO system model with mixed DACs. In Section 3, it performs the rate performance analysis. In addition, the weighted max–min power allocation scheme is conducted in Section 4. Moreover, Section 5 presents the numerical results and discussions to verify the derived results and finally, the conclusions are provided in Section 6.

Notation: Uppercase boldface and lowercase boldface letters are matrices and column vectors. $A^{*}$, $A^{T}$, and $A^{H}$ stand for the conjugate, transpose, and conjugate transpose of the matrix A, respectively. In addition, $I_{N}$ and $E\left\{\cdot \right\}$ stand for the N×N identity matrix and expectation operator. Moreover, $\left|\cdot \right|$ and $\left\|\cdot \right\|$ indicate absolute operator and Euclidean norm. Finally, n~CN(0,Ψ) represents a circularly symmetric complex Gaussian random vector with zero mean vector and covariance matrix Ψ, and $z\sim N(0,\delta^{2})$ denotes a real-value Gaussian random variable.

## 2. System Model

In this paper, a downlink cell-free massive MIMO system with mixed DACs is exhibited, where M APs serving K single-antenna users in the same resource blocks and each AP has *N* antennas. Both APs and users are randomly distributed within a D×D square area. To effectively reduce energy consumption and hardware cost, a mixed-DAC architecture is introduced. For each AP, its $N_{1}$ antennas are connected to perfect-resolution DACs, while the rest of the $N_{2}$ antennas are linked to low-resolution DACs, $N_{1}+N_{2}=N$. Furthermore, $\kappa =N_{1}/N$ denotes the proportion of the perfect DACs in the mixed-DAC architecture. Assuming the time division duplex (TDD) protocol is employed, then the downlink data transmission can use the uplink-acquired channel state information (CSI). Each coherence interval consists of two phases: uplink channel training and downlink data transmission [25].

Let $g_{m_{1}k}^{}\in {\mathbb{C}}^{N_{1}\times 1}$ be the channel gain from the k-th user to the *m*-th AP with perfect-resolution DACs and $g_{m_{2}k}^{}\in {\mathbb{C}}^{N_{2}\times 1}$ be the channel gain from the k-th user to the *m*-th AP with low-resolution DACs. Based on the general Rayleigh fading, the channel vectors $g_{m_{1}k}^{}$ and $g_{m_{2}k}^{}$ can be modeled as follows

(1) $$g_{m_{1}k}^{}=\beta_{mk}^{1/2}h_{m_{1}k},g_{m_{2}k}^{}=\beta_{mk}^{1/2}h_{m_{2}k},$$

where $\beta_{mk}^{},\forall m,\forall k$ is the large-scale fading factor, and $h_{m_{1}k}$, $h_{m_{2}k}$ represent the small-scale fading vectors. We assume that the entries of the small-scale fading are independent and identically distributed (*i.i.d.*) CN(0,1) random variables. Therefore, the channel from the k-th user to the m-th AP is given by $g_{mk}^{}={\left[g_{m_{1}k}^{T},g_{m_{2}k}^{T}\right]}^{T}\in {\mathbb{C}}^{N\times 1}$.

## 3. Uplink Channel Training

It is assumed that imperfect CSI is used. With the standard MMSE channel estimation technique, the downlink channel can be decomposed as

(2) $$g_{mk}^{}={\hat{g}}_{mk}^{}+e_{mk}^{},$$

where ${\hat{g}}_{mk}^{}$ and $e_{mk}^{}$ denote the estimated channel and the estimation error, respectively. The statistical characteristics of ${\hat{g}}_{mk}^{}$ and $e_{mk}^{}$ can be obtained from the following lemma.

**Lemma** **1.** *Assuming the orthogonal pilot sequences is less than the number of users, based on the property of MMSE, the characteristics of* ${\hat{g}}_{mk}^{}$ *and* $e_{mk}^{}$ *can be given as*

(3) $${\hat{g}}_{mk}^{}\sim CN\left(0,\gamma_{mk}\cdot I_{N}\right),$$

(4) $$e_{mk}^{}\sim CN\left(0,\left(\beta_{mk}^{}-\gamma_{mk}\right)\cdot I_{N}\right).$$

*In (3) and (4), the factor*$\gamma_{mk}$*is given by*

(5) $$\gamma_{mk}=\sqrt{\tau \rho_{p}}\beta_{mk}b_{mk}=\frac{\tau \rho_{p}\beta_{mk}^{2}}{\tau \rho_{p}\sum_{k^{\prime }=1}^{K}\beta_{mk^{\prime }}^{}\left|\varphi_{k}^{H}\varphi_{k^{\prime }}^{}\right|+1},$$

*where*$b_{mk}=\frac{\sqrt{\tau \rho_{p}}\beta_{mk}^{}}{\tau \rho_{p}\sum_{k^{\prime }=1}^{K}\beta_{mk^{\prime }}^{}{\left|\varphi_{k}^{H}\varphi_{k^{\prime }}^{}\right|}^{2}+1}$*,*τ*indicates the span of the training sequence,*$\rho_{p}$*denotes the normalized signal-to-noise ratio (SNR) for each pilot symbol, and*$\varphi_{k}^{}$*indicates the pilot sequence assigned to the k-th user,*${\left\|\varphi_{k}\right\|}^{2}=1$.

### 3.1. Downlink Data Transmission

In this phase, since the TDD protocol is advocated (the basic attribute of TDD is that the channel is reciprocal), the Aps will treat the estimated channel, ${\hat{g}}_{mk}^{}={\left[{\hat{g}}_{m_{1}k}^{T},{\hat{g}}_{m_{2}k}^{T}\right]}^{T}\in {\mathbb{C}}^{N\times 1}$, as the real channel for downlink data transmission. Thus, the precoded signal at the *m*-th AP with perfect-resolution DACs is formulated as

(6) $$x_{m_{1}}=\sqrt{\rho_{d}}\sum_{k=1}^{K}\sqrt{\eta_{mk}}{\hat{g}}_{m_{1}k}^{*}s_{k},$$

where $s_{k}$ is the signal for the *k*-th user satisfying $E\left\{{\left|s_{k}\right|}^{2}\right\}=1,\forall k$, and ${\hat{g}}_{m_{1}k}^{}\in {\mathbb{C}}^{N_{1}\times 1}$ denotes the channel from the *k*-th user to the *m*-th AP with perfect-resolution DACs. In addition, $\rho_{d}$ denotes the normalized downlink SNR and $\eta_{mk},\forall m,\forall k$ indicates the downlink power allocation coefficient. Similarly, the transmitted signal at the *m*-th AP with low-resolution DACs before quantization can be expressed as

(7) $$x_{m_{2}}=\sqrt{\rho_{d}}\sum_{k=1}^{K}\sqrt{\eta_{mk}}{\hat{g}}_{m_{2}k}^{*}s_{k},$$

where ${\hat{g}}_{m_{2}k}^{}\in {\mathbb{C}}^{N_{2}\times 1}$ is the channel vector from the *k*-th user to the *m*-th AP with low-resolution DACs.

Leveraging on the AQNM (in reality, the AQNM has been widely adopted in the quantized MIMO system, due to the fact it can provide accurate estimation under the low- and medium-SNR scenarios [20,23]), the version of the quantized signal at *m*-th AP with low-resolution DACs is

(8) $${\tilde{x}}_{m_{2}}=Q\left(x_{m_{2}}\right)=\alpha_{m}\sqrt{\rho_{d}}\sum_{k=1}^{K}\sqrt{\eta_{mk}}{\hat{g}}_{m_{2}k}^{*}s_{k}+{\tilde{n}}_{m_{2}},$$

where Q(⋅) is a linear quantization function, $\alpha_{m}$ denotes the linear gain shown in the following Table 1 (for $b_{m}>5,\forall m$, in general, the quantization coefficient can be approximated as $\alpha_{m}\approx \pi \sqrt{3}/2\times 2^{-2b_{m}}$), and ${\tilde{n}}_{m_{2}}$ means the quantization noise vector.

The covariance matrix of ${\tilde{n}}_{m_{2}}$ is given by [26]

(9) $$\begin{array}{cc} R_{{\tilde{n}}_{m_{2}}} & =\alpha_{m}\left(1-\alpha_{m}\right)\mathrm{diag}\left(E\left\{x_{m_{2}}x_{m_{2}}^{H}\right\}\right)\\ & =\alpha_{m}\left(1-\alpha_{m}\right)\rho_{d}\sum_{k=1}^{K}\eta_{mk}\gamma_{mk}I_{N_{2}}. \end{array}$$

According to (6) and (8), the overall transmitted signal at the m-th AP can be expressed as

(10) $$x_{m}=\left[\begin{array}{c} x_{m_{1}}\\ {\tilde{x}}_{m_{2}} \end{array}\right]=\left[\begin{array}{l} \sqrt{\rho_{d}}\sum_{k=1}^{K}\sqrt{\eta_{mk}}{\hat{g}}_{m_{1}k}^{*}s_{k}\\ \alpha_{m}\sqrt{\rho_{d}}\sum_{k=1}^{K}\sqrt{\eta_{mk}}{\hat{g}}_{m_{2}k}^{*}s_{k}+{\tilde{n}}_{m_{2}} \end{array}\right].\;$$

To satisfy the following power constraint at each AP, we have $E\left\{{\left\|x_{m}\right\|}^{2}\right\}\leq \rho_{d}.$ Then, the power of the transmitted signal is

(11) $$\begin{array}{ll} E\left\{{\left\|x_{m}\right\|}^{2}\right\} & =E\left\{{\left\|x_{m_{1}}\right\|}^{2}+{\left\|{\tilde{x}}_{m_{2}}\right\|}^{2}\right\}\\ & =N_{1}\rho_{d}\sum_{k=1}^{K}\eta_{mk}\gamma_{mk}+N_{2}\rho_{d}\alpha_{m}^{}\sum_{k=1}^{K}\eta_{mk}\gamma_{mk}\\ & =\left(N_{1}+\alpha_{m}^{}N_{2}\right)\rho_{d}\sum_{k=1}^{K}\eta_{mk}\gamma_{mk}. \end{array}$$

Therefore, the constraint condition can be given as

(12) $$\sum_{k=1}^{K}\eta_{mk}\gamma_{mk}\leq \frac{1}{N_{1}+N_{2}\alpha_{m}^{}},\forall m.$$

In the next subsection, the rate performance for the considered downlink cell-free massive MIMO system with mixed DACs is conducted.

### 3.2. Rate Performance Analysis

With the overall transmitted signal $x_{m}$, then the received signal at the *k*-th user can be written as

(13) $$\begin{array}{ll} r_{k} & =\sum_{m=1}^{M}g_{mk}^{T}x_{m}+w_{k}=\sum_{m=1}^{M}g_{m_{1}k}^{T}x_{m_{1}}+\sum_{m=1}^{M}\alpha_{m}g_{m_{2}k}^{T}x_{m_{2}}+\sum_{m=1}^{M}g_{m_{2}k}^{T}{\tilde{n}}_{m_{2}}+w_{k}\\ & =\sqrt{\rho_{d}}\sum_{m=1}^{M}g_{m_{1}k}^{T}\sqrt{\eta_{mk}}{\hat{g}}_{m_{1}k}^{*}s_{k}+\sum_{m=1}^{M}g_{m_{2}k}^{T}{\tilde{n}}_{m_{2}}+\sqrt{\rho_{d}}\sum_{k^{\prime }\neq k}^{K}\sum_{m=1}^{M}\sqrt{\eta_{mk^{\prime }}}g_{m_{1}k}^{T}{\hat{g}}_{m_{1}k^{\prime }}^{*}s_{k^{\prime }}\\ & +\sqrt{\rho_{d}}\sum_{k^{\prime }\neq k}^{K}\sum_{m=1}^{M}\alpha_{m}\sqrt{\eta_{mk^{\prime }}}g_{m_{2}k}^{T}{\hat{g}}_{m_{2}k^{\prime }}^{*}s_{k^{\prime }}+\sqrt{\rho_{d}}\sum_{m=1}^{M}\alpha_{m}\sqrt{\eta_{mk}}g_{m_{2}k}^{T}{\hat{g}}_{m_{2}k}^{*}s_{k}+w_{k}, \end{array}$$

where $w_{k}\sim CN(0,1)$ denotes the additive white Gaussian noise (AWGN) at the *k*-th user. With this signal, the ergodic rate of the *k*-th user can be given as

(14) $$\begin{array}{c} R_{k}^{\mathrm{erg}}=E\left\{\log_{2}\left(1+\frac{\rho_{d}{\left|\sum_{m=1}^{M}g_{m_{1}k}^{T}\sqrt{\eta_{mk}}{\hat{g}}_{m_{1}k}^{*}+\sum_{m=1}^{M}\alpha_{m}\sqrt{\eta_{mk}}g_{m_{2}k}^{T}{\hat{g}}_{m_{2}k}^{*}\right|}^{2}}{\rho_{d}\sum_{k^{\prime }\neq k}^{K}{\left|\sum_{m=1}^{M}\sqrt{\eta_{mk^{\prime }}}g_{m_{1}k}^{T}{\hat{g}}_{m_{1}k^{\prime }}^{*}+\sum_{m=1}^{M}\alpha_{m}\sqrt{\eta_{mk^{\prime }}}g_{m_{2}k}^{T}{\hat{g}}_{m_{2}k^{\prime }}^{*}\right|}^{2}+{\left|\sum_{m=1}^{M}g_{m_{2}k}^{T}{\tilde{n}}_{m_{2}}\right|}^{2}+1}\right)\right\}. \end{array}$$

By using the use-and-then-forget (UatF) technique as given in [19], the received signal at the *k*-th user can be rewritten as

(15) $$r_{k}=\underset{\mathrm{desired}\;\mathrm{signal}}{\underset{︸}{\sqrt{\rho_{d}}E\left\{\sum_{m=1}^{M}\sqrt{\eta_{mk}}\left(g_{m_{1}k}^{T}{\hat{g}}_{m_{1}k}^{*}+\alpha_{m}g_{m_{2}k}^{T}{\hat{g}}_{m_{2}k}^{*}\right)\right\}}}s_{k}+\underset{\mathrm{additive}\;\mathrm{noise}}{\underset{︸}{{\tilde{w}}_{k},}}$$

where ${\tilde{w}}_{k}$ indicates the additive noise denoted by

(16) $$\begin{array}{ll} {\tilde{w}}_{k}= & \underset{\mathrm{beamforming}\;\mathrm{gain}\;\mathrm{uncertaintyfor}\;\mathrm{perfect}\;\mathrm{DACs}}{\underset{︸}{\sqrt{\rho_{d}}\sum_{m=1}^{M}\left(g_{m_{1}k}^{T}\sqrt{\eta_{mk}}{\hat{g}}_{m_{1}k}^{*}-E\left\{g_{m_{1}k}^{T}\sqrt{\eta_{mk}}{\hat{g}}_{m_{1}k}^{*}\right\}\right)s_{k}}}+\underset{\mathrm{additive}\;\mathrm{noise}}{\underset{︸}{w_{k}}}\\ & +\underset{\mathrm{beamforming}\;\mathrm{gain}\;\mathrm{uncertainty}\;\mathrm{for}\;\mathrm{coarse}\;\mathrm{DACs}}{\underset{︸}{\sqrt{\rho_{d}}\sum_{m=1}^{M}\alpha_{m}\sqrt{\eta_{mk}}\left(g_{m_{2}k}^{T}{\hat{g}}_{m_{2}k}^{*}-E\left\{g_{m_{2}k}^{T}{\hat{g}}_{m_{2}k}^{*}\right\}\right)s_{k}}}+\underset{\mathrm{quantization}\;\mathrm{noise}\;\mathrm{for}\;\mathrm{coarse}\;\mathrm{DACs}}{\underset{︸}{\sum_{m=1}^{M}g_{m_{2}k}^{T}{\tilde{n}}_{m_{2}}}}\\ & +\underset{\mathrm{inter}-\mathrm{user}\;\mathrm{interference}\;\mathrm{for}\;\mathrm{coarse}\;\mathrm{DACs}}{\underset{︸}{\sqrt{\rho_{d}}\sum_{k^{\prime }\neq k}^{K}\sum_{m=1}^{M}\alpha_{m}\sqrt{\eta_{mk^{\prime }}}g_{m_{2}k}^{T}{\hat{g}}_{m_{2}k^{\prime }}^{*}s_{k^{\prime }}}}+\underset{\mathrm{inter}-\mathrm{user}\;\mathrm{interference}\;\mathrm{for}\;\mathrm{perfect}\;\mathrm{DACs}}{\underset{︸}{\sqrt{\rho_{d}}\sum_{k^{\prime }\neq k}^{K}\sum_{m=1}^{M}\sqrt{\eta_{mk^{\prime }}}g_{m_{1}k}^{T}{\hat{g}}_{m_{1}k^{\prime }}^{*}s_{k^{\prime }}}}. \end{array}$$

With the above consideration, the corresponding downlink rate can be shown as

(17) $$R_{k}=\log_{2}\left(1+\frac{{\mathrm{DS}}_{k}}{{\mathrm{BU}}_{k}+\sum_{k^{\prime }\neq k}^{K}{\mathrm{UI}}_{kk^{\prime }}+{\mathrm{QI}}_{k}+1}\right),$$

where ${\mathrm{DS}}_{k}$, ${\mathrm{BU}}_{k}$, ${\mathrm{UI}}_{kk^{\prime }}$, and ${\mathrm{QI}}_{k}$ represent the desired signal, the beamforming uncertainty gain for perfect and low-resolution DACs, the inter-user interference for perfect and low-resolution DACs, and the quantization noise interference for low-resolution DACs, which can, respectively, be denoted as follows

(18) $${\mathrm{DS}}_{k}={\left|\sqrt{\rho_{d}}\sum_{m=1}^{M}\sqrt{\eta_{mk}}E\left\{g_{m_{1}k}^{T}{\hat{g}}_{m_{1}k}^{*}+\alpha_{m}g_{m_{2}k}^{T}{\hat{g}}_{m_{2}k}^{*}\right\}\right|}^{2},$$

(19) $$\begin{array}{ll} {\mathrm{BU}}_{k} & =E\left\{\rho_{d}{\left|\sum_{m=1}^{M}\sqrt{\eta_{mk}}\left(g_{m_{1}k}^{T}{\hat{g}}_{m_{1}k}^{*}-E\left\{g_{m_{1}k}^{T}{\hat{g}}_{m_{1}k}^{*}\right\}\right)\right|}^{2}\right\}\\ & +E\left\{\rho_{d},{\left|\sum_{m=1}^{M}\alpha_{m}\sqrt{\eta_{mk}}\left(g_{m_{2}k}^{T}{\hat{g}}_{m_{2}k}^{*}-E\left\{g_{m_{2}k}^{T}{\hat{g}}_{m_{2}k}^{*}\right\}\right)\right|}^{2}\right\}, \end{array}$$

(20) $${\mathrm{UI}}_{kk^{\prime }}=E\left\{{\left|\sqrt{\rho_{d}}\sum_{m=1}^{M}\sqrt{\eta_{mk^{\prime }}}g_{m_{1}k}^{T}{\hat{g}}_{m_{1}k^{\prime }}^{*}\right|}^{2}\right\}+E\left\{{\left|\sqrt{\rho_{d}}\sum_{m=1}^{M}\alpha_{m}\sqrt{\eta_{mk^{\prime }}}g_{m_{2}k}^{T}{\hat{g}}_{m_{2}k^{\prime }}^{*}\right|}^{2}\right\},$$

(21) $${\mathrm{QI}}_{k}=E\left\{{\left|\sum_{m=1}^{M}g_{m_{2}k}^{T}{\tilde{n}}_{m_{2}}\right|}^{2}\right\}.$$

**Theorem** **1.** *For the considered downlink cell-free massive MIMO system with mixed DACs, the achievable rate expression of the k-th user is*

(22) $$R_{k}=\log_{2}\left(1+\frac{\rho_{d}{\left|\sum_{m=1}^{M}\sqrt{\eta_{mk}}\left(N_{1}\gamma_{mk}+\alpha_{m}N_{2}\gamma_{mk}\right)\right|}^{2}}{\rho_{d}\sum_{k^{\prime }\neq k}^{K}{\left|{\tilde{\varphi }}_{k}^{H}{\tilde{\varphi }}_{k^{\prime }}^{}\right|}^{2}\left(N_{1}^{2}{\left(\sum_{m=1}^{M}\sqrt{\eta_{mk^{\prime }}}\gamma_{mk^{\prime }}\frac{\beta_{mk}}{\beta_{mk^{\prime }}}\right)}^{2}+\Delta_{k}\right)+\Psi_{k}}\right),$$

*where*

(23) $$\begin{array}{ll} \Delta_{k}= & N_{2}^{2}{\left(\sum_{m=1}^{M}\alpha_{m}\sqrt{\eta_{mk^{\prime }}}\gamma_{mk^{\prime }}\frac{\beta_{mk}}{\beta_{mk^{\prime }}}\right)}^{2}\\ & +2\cdot \mathrm{Real}\left(N_{1},N_{2},\sum_{m=1}^{M}\sum_{n=1}^{M}\left(\alpha_{m}\sqrt{\eta_{mk^{\prime }}}\gamma_{mk^{\prime }}\frac{\beta_{mk}}{\beta_{mk^{\prime }}}\right)\left(\sqrt{\eta_{nk^{\prime }}}\gamma_{nk^{\prime }}\frac{\beta_{nk}}{\beta_{nk^{\prime }}}\right)\right), \end{array}$$

(24) $$\Psi_{k}=\rho_{d}\sum_{m=1}^{M}\left(N_{1}+\alpha_{m}N_{2}\right)\beta_{mk}\sum_{k^{\prime }=1}^{K}\sqrt{\eta_{mk^{\prime }}}\gamma_{mk^{\prime }}+1.$$

**Proof** **of** **Theorem** **1.** See Appendix A. □

Based on the above theorem, the sum rate of the considered cell-free massive MIMO systems is

(25) $$R^{\mathrm{sum}}=\sum_{k=1}^{K}R_{k}.$$

In order to cater to the diverse rate requirements of multiple users in future scenarios, the weighted max–min power allocation algorithm is presented, in which several emergencies such as telemedicine, driverless, and real-time operations often require high-priority in contrast to the others (note that a user usually enjoying high-priority expects a smaller weighting factor and vice versa). Motived by this consideration, a weighted max–min power allocation scheme is presented in the next section.

## 4. Weighed Max–Min Power Allocation Scheme

Based on the derived closed-form rate expression (22), mathematically, the proposed weighted max–min power allocation scheme can be formulated as

(26) $$P_{1}:\underset{\left\{\eta_{k}\right\}}{\max}\;\underset{\forall k}{\min}\;\omega_{k}R_{k}$$

(27) $$s.t.\sum_{k=1}^{K}\eta_{mk}\gamma_{mk}\leq \frac{1}{N_{1}+N_{2}\alpha_{m}},\forall m$$

(28) $$\eta_{mk}\geq 0,\forall k,\forall m$$

where $\omega_{k}$ denotes the weighting factor, $0\leq \omega_{k}\leq 1,\forall k$, and $\eta_{k}={\left[\eta_{1k}^{}\eta_{2k}^{}\ldots \eta_{Mk}^{}\right]}^{T}$. By defining $\varepsilon_{mk}=\eta_{mk}^{1/2}$ and introducing several slack variables t, $p_{k^{\prime }k}$, $q_{k^{\prime }k}$, $z_{k^{\prime }k}$, and $v_{m}$, $P_{1}$ can be reformulated as the following

(29) $$P_{2}:\underset{\left\{t,\varepsilon_{k},p_{k^{\prime }k},q_{k^{\prime }k},z_{k^{\prime }k},v_{m}\right\}}{\max}\underset{\forall k}{\min}\;t$$

(30) $$s.t.t\leq \omega_{k}\frac{{\left|\sum_{m=1}^{M}\varepsilon_{mk}\left(N_{1}\gamma_{mk}+\alpha_{m}N_{2}\gamma_{mk}\right)\right|}^{2}}{\sum_{k^{\prime }\neq k}^{K}{\left|\varphi_{k}^{H}\varphi_{k^{\prime }}\right|}^{2}z_{k^{\prime }k}^{2}+\sum_{m=1}^{M}\beta_{mk}v_{m}+\frac{1}{\rho_{d}}},\forall k$$

(31) $$\sum_{m=1}^{M}\varepsilon_{mk^{\prime }}\gamma_{mk^{\prime }}\frac{\beta_{mk}}{\beta_{mk^{\prime }}}\leq \frac{p_{k^{\prime }k}}{N_{1}^{}},\forall k^{\prime }\neq k$$

(32) $$\sum_{m=1}^{M}\alpha_{m}\varepsilon_{mk^{\prime }}\gamma_{mk^{\prime }}\frac{\beta_{mk}}{\beta_{mk^{\prime }}}\leq \frac{q_{k^{\prime }k}}{N_{2}^{}},\forall k^{\prime }\neq k$$

(33) $$\sum_{k^{\prime }=1}^{K}\varepsilon_{mk^{\prime }}\gamma_{mk^{\prime }}\leq \frac{v_{m}}{N_{1}+\alpha_{m}N_{2}},\forall m$$

(34) $$\sum_{k=1}^{K}\varepsilon_{mk}^{2}\gamma_{mk}\leq \frac{1}{N_{1}+N_{2}\alpha_{m}},\forall m$$

(35) $$z_{k^{\prime }k}\leq p_{k^{\prime }k}+q_{k^{\prime }k},\forall k^{\prime }\neq k$$

(36) $$\eta_{mk}\geq 0,\forall m,\forall k$$

As the objective function (29) can be proved to be quasi-concave [3], the optimization problem can be solved by the following bisection Algorithm 1.
**Algorithm 1** Bisection Algorithm for Solving $P_{2}$**Input**: $M,K,N,N_{1},\tau ,\rho_{d},\gamma_{mk}.$**Output**: $\varepsilon_{k},\forall k,\forall m$(1) Initialize the lower value $r_{l}$ and the upper bound $r_{u}$. Give the tolerance δ>0.(2) **While**
$r_{u}-r_{l}\leq \delta$(3) **Set**
$r=\left(r_{l}+t_{u}\right)/2$ and solve the following feasible problem ${\tilde{P}}_{2}$.(4) If ${\tilde{P}}_{2}$ is feasible, set $r_{l}=r$, go to step 2, else set $r_{u}=r$.(5) **end If**(6) **end While**

At each iteration step, the following convex feasibility problem should be determined [29,32]

(37) $${\tilde{P}}_{2}:\left\|s_{k}\right\|\leq \frac{\omega_{k}^{1/2}\sum_{m=1}^{M}\varepsilon_{mk}\left(N_{1}\gamma_{mk}+\alpha_{m}N_{2}\gamma_{mk}\right)}{\sqrt{t}},\forall k$$

(38) s.t.(31), (32),(33),(34), (35),(36).

where $s_{k}={\left[s_{k1}^{T}I_{-k}s_{k2}^{T}\frac{1}{\sqrt{\rho_{d}}}\right]}^{T}$, $s_{k1}^{}={\left[\varphi_{k}^{H}\varphi_{1}z_{1k}\ldots \varphi_{k}^{H}\varphi_{K}z_{Kk}\right]}^{T}$, and $s_{k2}^{}={\left[\sqrt{\beta_{1k}}v_{1}\ldots \sqrt{\beta_{Mk}}v_{M}\right]}^{T}$.

## 5. Numerical Results

In this section, the simulation results and discussions are conducted. We assume all APs and users are uniformly and randomly distributed within a D×D square area.

### 5.1. Simulation Parameters

Followed by [3,6], the large-scale fading coefficient is generated by

(39) $$\beta_{mk}={\mathrm{PL}}_{mk}\cdot 10^{\frac{\sigma_{\mathrm{sh}}z_{mk}}{10}},$$

where ${\mathrm{PL}}_{mk}$ and $10^{\frac{\sigma_{\mathrm{sh}}z_{mk}}{10}}$ reflect the effects of pathloss and shadow fading, $z_{mk}\sim N(0,1)$. Specifically, we use the three-slope model to describe ${\mathrm{PL}}_{mk}$ as in [14,21]

(40) $${\mathrm{PL}}_{mk}=\left\{\begin{array}{l} -L-35\mathrm{lg}\left(d_{mk}\right),\mathrm{if}d_{mk}>d_{1}\\ -L-15\mathrm{lg}\left(d_{1}\right)-20\mathrm{lg}\left(d_{mk}\right),\mathrm{if}d_{0}<d_{mk}\leq d_{1}\\ -L-15\mathrm{lg}\left(d_{1}\right)-20\mathrm{lg}\left(d_{0}\right),\mathrm{if}d_{mk}\leq d_{0}, \end{array}\right.$$

where L is a constant depending on the carrier frequency, $d_{0}$ and $d_{1}$ are the reference distances, and $d_{mk}$ represents the geographical distance between the *m*-th AP and the *k*-th user. Unless otherwise specified, the simulation parameters are given in Table 2.

### 5.2. Results and Discussions

In this section, the numerical simulations are provided to validate the previous theoretical analysis. We assume that all APs and users are randomly distributed in the wrapped-around area to imitate an infinite communication environment.

**Figure 1:**
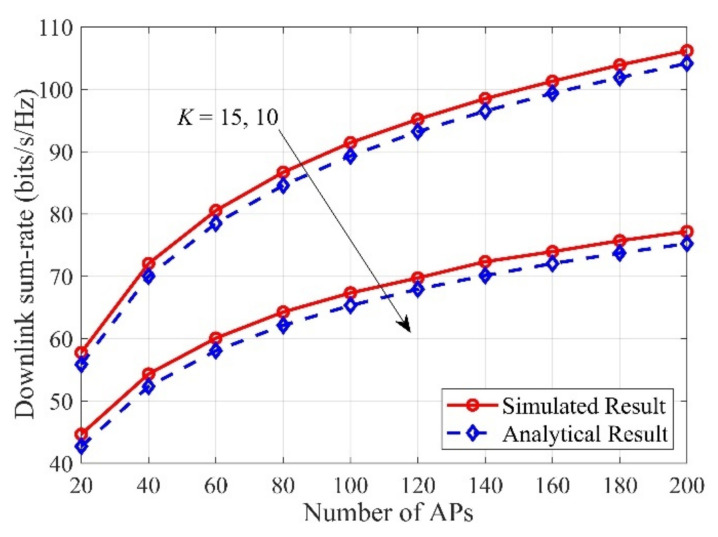
Downlink sum-rate versus the number of access points (Aps) with different numbers of users, with M=60, N=10.

First, to verify the correctness of the theoretical deduction, based on the genie-assisted “Simulated Result” in (14) and the “Analytical Result” in (22), in Figure 1, a comparison about downlink sum-rate against the number of APs is presented. Typically, the “Simulated Result” is generated by using the Monte-Carlo method by averaging $10^{4}$ independent channel realizations. As can be readily observed, the “Simulated Result” exactly matches the “Analytical Result” for all considered cases, which confirms that our derived analytical results are effective and correct. In addition, we can easily find that the downlink sum rate grows with the number of APs. The reason for this observation is that large numbers of distributed APs can bring huge antenna gains to the system.

**Figure 2:**
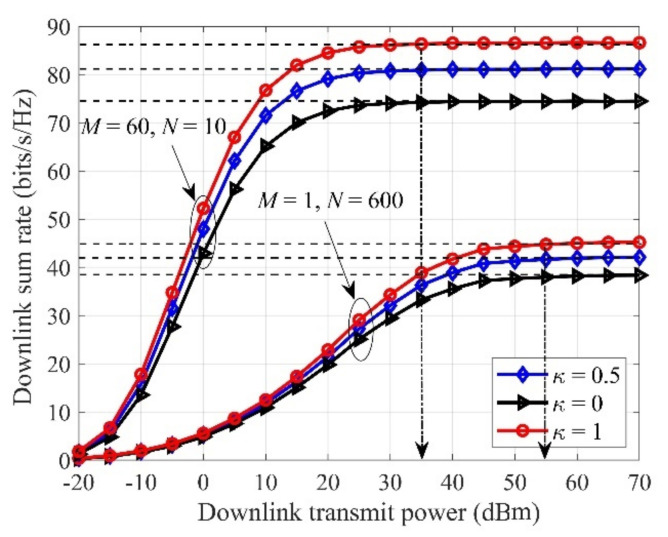
Downlink sum-rate versus the transmitted power with different antenna configurations, with MN=600, K=10..

In Figure 2, we present the sum-rate against different κ for MN=600, K=10. Typically, “M=1, N=600” represents the “traditional collocated massive MIMO”, whilst the legend “M=60, N=10” corresponds to the “cell-free massive MIMO”. It is clear that the achievable downlink sum rate will saturate to a certain bound in the high-power regime, since it is jointly subjected to multi-user interference, pilot contamination, and quantization noise. For these two configurations, the rate with “M = 60, N = 10” is superior to “M=1, N=600”. We find that the required minimum transmitted power is different to achieve the maximum rate for different configurations. For example, the minimum transmitted power is 35 dBm and 55 dBm for κ=1.

**Figure 3:**
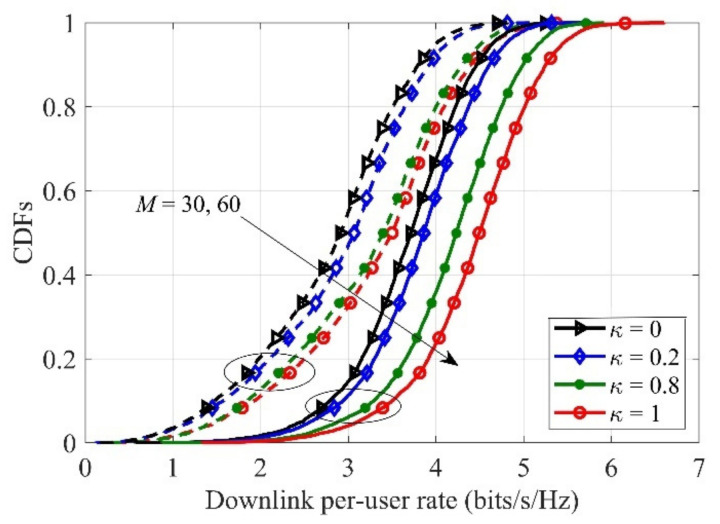
Cumulative distribution functions (CDFs) of the downlink per-user rate for different users, with K= 10, N = 10..

In Figure 3, the cumulative distribution functions (CDFs) of the downlink per-user rate are given, with N=10 and K=10. As we see, the pure low-resolution DACs suffer from the worst rate because the quantization distortion will bring great performance loss. Quantitatively speaking, the 5%-outage rate of “κ=1” is 2.93 bits/s/Hz, which is improved by factors of 1.33 and 1.18 compared with the cases “κ=0.2” and “κ=0.8”, respectively. Therefore, increasing “κ” can effectively boost the achievable downlink sum rate.

**Figure 4:**
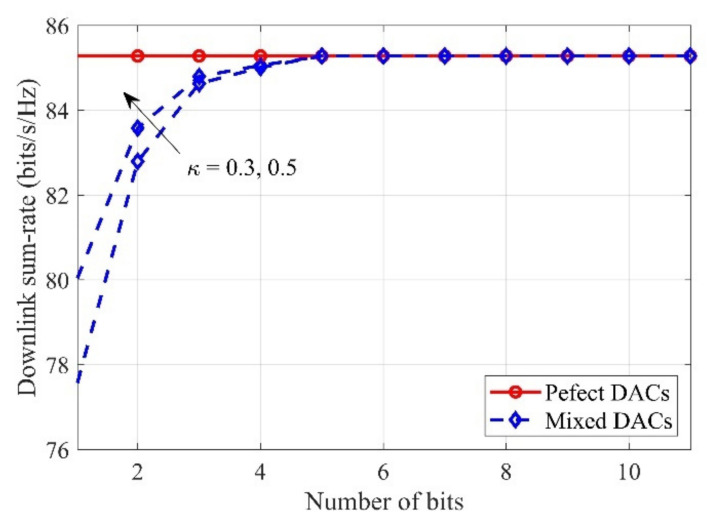
Downlink sum-rate versus the number of bits for different κ, with M=60, N=10, K=10.

Figure 4 investigates the downlink sum-rate for the number of DAC bits for different κ. It is clear to see that when the DAC bit is less than 5 bit, increasing $b_{m},\forall m$ can effectively increase the downlink rate. However, when $b_{m}\geq 5$, the system performance will saturate. This means that, in this regime, 5-bit DACs can be used to replace perfect-resolution DACs without any performance loss. In addition, it can be seen that increasing the proportion of the perfect DACs can effectively improve the system rate.

**Figure 5:**
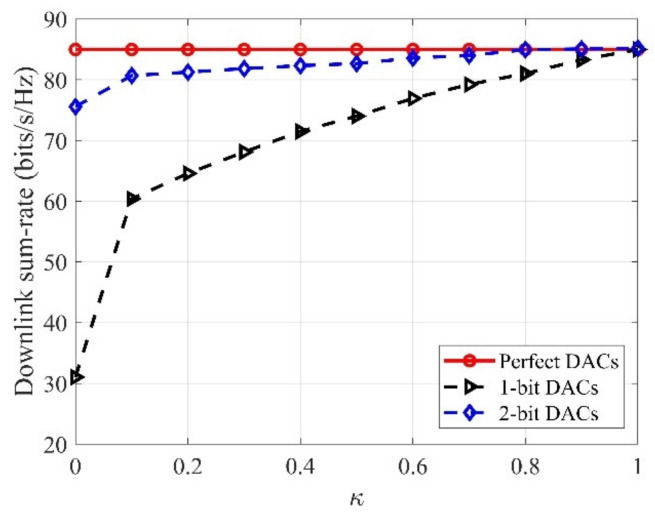
Downlink sum-rate versus the κ for different quantization bits, with M=60, N=10, K=10.

In Figure 5, the downlink sum-rate versus the κ for different quantization bits is described, with M=60, N=10, K=10. From Figure 5 we see that, for the low-resolution DACs, the downlink sum-rate raises as the κ increases, especially for 1-bit DACs. The reason is that with the increased κ, the proportion of the perfect DACs is raised, and then the downlink rate of the system can be greatly improved.

**Figure 6:**
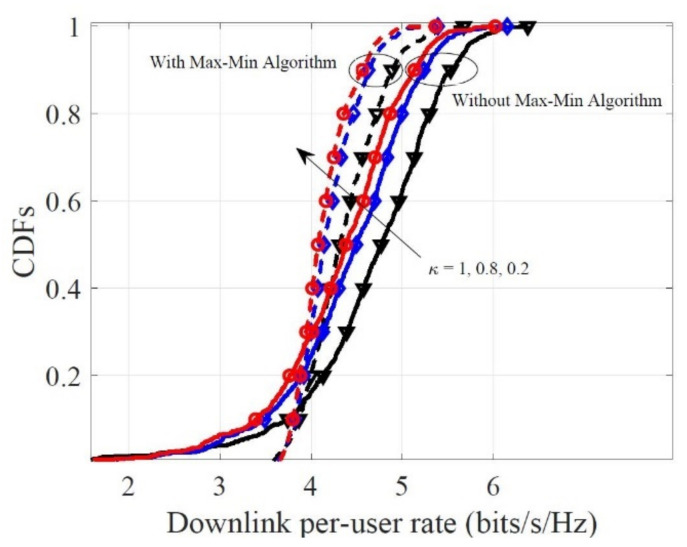
CDFs of the downlink per-user rate for the different κ, with M=60, N=10, K=10.

Last but not least, Figure 6 investigates the impact of the κ for the downlink per-user rate for the general fairness principle ($\omega_{k}=1,\forall k$), with M=60, N=10, K=10. Along with the κ increase, the downlink per-user rate can be sharply enhanced and the gaps between different curves are decreased. The results show that when the system uses low-resolution quantization, increasing κ contributes to raising the system sum rate. Focusing on the general fairness principle, the achievable downlink rate is more concentrated around a certain value compared with the case without the max–min algorithm. This confirms that the proposed algorithm can guarantee uniform QoS.

Finally, we verify the validity of the proposed weighted max–min power allocation algorithm in Table 3, assuming that the users k,∀k≤3 enjoy high priority, and their weighting factors are $\omega_{k}=0.5,\forall k\leq 3$, while the remaining users are $\omega_{k}=1,4\leq k\leq 10$. The presented results show that the proposed weighted max–min scheme can guarantee the high-priority users have a higher rate, and the remaining users can have the uniform rate. The results have significant value for the high-priority users in future emergencies.

## 6. Conclusions

In this paper, we explored the downlink cell-free massive MIMO systems with mixed DACs. With the aid of the AQNM and conjugate beamforming receiver, the tight closed-form of the downlink rate was derived. Based on the derived results, the effects of system parameters such as the number of APs, the downlink transmitted power, the number of quantization bits, and the proportion of perfect DACs were investigated. This shows that when the quantization bits reach 5 bits, the system performance will saturate the uniform value as in the case of perfect DACs. Lastly, to provide a high rate for the high-priority users in future emerging situations, whilst the remaining users have uniform rates, an effective weighted max–min algorithm is carried out. The numerical results and discussions are provided to verify the effectiveness of the proposed algorithm.

## Figures and Tables

**Table 1:** Quantization Bits $b_{m}$ for Coeficient $\alpha_{m}$.

$b_{m}$	1	2	3	4	5
$\alpha_{m}$	0.6366	0.8825	0.96546	0.990503	0.997501

**Table 2:** Simulation Parameters.

Parameter	Value
*B*	20 MHz
*T*	200
$\rho_{p},\rho_{d}$	100, 200 mW
$d_{0},d_{1},D$	10, 50, 1000 m

**Table 3:** The Achievable Per-User Rate for proposed algorithm, for 1⩽n⩽3, $\omega_{k}=0.5$, else $\omega_{k}=1$.

Achievable Per-User Rate (bits/s/Hz)										
**Quantization Bits**	$R_{1}$	$R_{2}$	$R_{3}$	$R_{4}$	$R_{5}$	$R_{6}$	$R_{7}$	$R_{8}$	$R_{9}$	$R_{10}$
DAC = 1	5.07	5.07	5.07	4.15	4.15	4.15	4.15	4.15	4.15	4.15
DAC = 3	5.91	5.91	5.91	4.96	4.96	4.96	4.96	4.96	4.96	4.96
DAC = 10	6.02	6.02	6.02	5.06	5.06	5.06	5.06	5.06	5.06	5.06

## Data Availability

Not applicable.

## Appendix A

**Proof** **of** **Theorem** **1.** To derive the closed-form expression for the considered downlink rate expressed in (17), we need to derive ${\mathrm{DS}}_{k}$, ${\mathrm{BU}}_{k}$, ${\mathrm{UI}}_{kk^{\prime }}$, and ${\mathrm{QI}}_{k}$, respectively. □

(1) Compute ${\mathrm{DS}}_{k}$: Due to the fact that $g_{mk}={\hat{g}}_{mk}+e_{mk}$, and ${\hat{g}}_{mk}$ is independent $e_{mk}$ under MMSE estimation theory, therefore, we have

(A1) $$\begin{array}{l} \sum_{m=1}^{M}E\left\{g_{m_{1}k}^{T}{\hat{g}}_{m_{1}k}^{*}+\alpha_{m}g_{m_{2}k}^{T}{\hat{g}}_{m_{2}k}^{*}\right\}\\ =\sum_{m=1}^{M}E\left\{\left({\hat{g}}_{m_{1}k}^{T}+e_{m_{1}k}^{T}\right){\hat{g}}_{m_{1}k}^{*}+\alpha_{m}\left({\hat{g}}_{m_{2}k}^{T}+e_{m_{2}k}^{T}\right){\hat{g}}_{m_{2}k}^{*}\right\}\\ =\sum_{m=1}^{M}\left(N_{1}\gamma_{mk}+\alpha_{m}N_{2}\gamma_{mk}\right). \end{array}$$

(2) Compute ${\mathrm{BU}}_{k}$: With the theory of probability and statistics, we have the following fact that the variance of a sum of independent random variables is equal to the sum of the variances, we have

(A2) $$\begin{array}{ll} BU_{k} & =E\left\{\rho_{d}{\left|\sum_{m=1}^{M}\sqrt{\eta_{mk}}\left(g_{m_{1}k}^{T}{\hat{g}}_{m_{1}k}^{*}-E\left\{g_{m_{1}k}^{T}{\hat{g}}_{m_{1}k}^{*}\right\}\right)\right|}^{2}\right\}\\ & +E\left\{\rho_{d}{\left|\sum_{m=1}^{M}\alpha_{m}\sqrt{\eta_{mk}}\left(g_{m_{2}k}^{T}{\hat{g}}_{m_{2}k}^{*}-E\left\{g_{m_{2}k}^{T}{\hat{g}}_{m_{2}k}^{*}\right\}\right)\right|}^{2}\right\}\\ & =\rho_{d}\sum_{m=1}^{M}\eta_{mk}\cdot E\left\{{\left|g_{m_{1}k}^{T}{\hat{g}}_{m_{1}k}^{*}+\alpha_{m}g_{m_{2}k}^{T}{\hat{g}}_{m_{2}k}^{*}\right|}^{2}\right.-\left.{\left|E\left\{g_{m_{1}k}^{T}{\hat{g}}_{m_{1}k}^{*}+\alpha_{m}g_{m_{2}k}^{T}{\hat{g}}_{m_{2}k}^{*}\right\}\right|}^{2}\right\}. \end{array}$$

The first part $E\left\{{\left|g_{m_{1}k}^{T}{\hat{g}}_{m_{1}k}^{*}+\alpha_{m}g_{m_{2}k}^{T}{\hat{g}}_{m_{2}k}^{*}\right|}^{2}\right\}$ can be rewritten as follows

(A3) $$\begin{array}{l} E\left\{{\left|g_{m_{1}k}^{T}{\hat{g}}_{m_{1}k}^{*}+\alpha_{m}g_{m_{2}k}^{T}{\hat{g}}_{m_{2}k}^{*}\right|}^{2}\right\}\\ =E\left\{\alpha_{m}^{2}{\left|g_{m_{2}k}^{T}{\hat{g}}_{m_{2}k}^{*}\right|}^{2}+{\left|g_{m_{1}k}^{T}{\hat{g}}_{m_{1}k}^{*}\right|}^{2}\right.\left.+2\alpha_{m}\cdot \mathrm{Real}\left(g_{m_{1}k}^{T}{\hat{g}}_{m_{1}k}^{*}g_{m_{2}k}^{T}{\hat{g}}_{m_{2}k}^{*}\right)\right\}\\ \overset{\left(a\right)}{=}\;\alpha_{m}^{2}\left(N_{2}\gamma_{mk}\left(\beta_{mk}^{}-\gamma_{mk}^{}\right)+N_{2}\left(N_{2}+1\right)\gamma_{mk}^{2}\right)\\ +N_{1}\gamma_{mk}\left(\beta_{mk}^{}-\gamma_{mk}^{}\right)+N_{1}\left(N_{1}+1\right)\gamma_{mk}^{2}+2\alpha_{m}N_{1}N_{2}\gamma_{mk}^{2}\\ =\left(N_{1}+\alpha_{m}^{2}N_{2}\right)\gamma_{mk}\beta_{mk}^{}+2\alpha_{m}N_{1}N_{2}\gamma_{mk}^{2}+\left(N_{1}^{2}+\alpha_{m}^{2}N_{2}^{2}\right)\gamma_{mk}^{2}, \end{array}$$

where (*a*) follows the fact that $E\left\{{\left\|{\hat{g}}_{m_{1}k}^{}\right\|}^{2}\right\}=N_{1}\gamma_{mk}$, $E\left\{{\left\|{\hat{g}}_{m_{1}k}^{}\right\|}^{4}\right\}=N_{1}\left(N_{1}+1\right)\gamma_{mk}^{2}$, and $E\left\{{\left|{\hat{g}}_{m_{1}k}^{H}e_{m_{1}k}\right|}^{2}\right\}=N_{1}\gamma_{mk}^{}\left(\beta_{mk}^{}-\gamma_{mk}^{}\right)$.

For the second part $E\left\{g_{m_{1}k}^{T}{\hat{g}}_{m_{1}k}^{*}+\alpha_{m}g_{m_{2}k}^{T}{\hat{g}}_{m_{2}k}^{*}\right\}$, along with the same lines, we have

(A4) $$E\left\{g_{m_{1}k}^{T}{\hat{g}}_{m_{1}k}^{*}+\alpha_{m}g_{m_{2}k}^{T}{\hat{g}}_{m_{2}k}^{*}\right\}=N_{1}^{}\gamma_{mk}^{}+\alpha_{m}N_{2}^{}\gamma_{mk}^{}.$$

Combining (A3) and (A4), we have

(A5) $$\begin{array}{l} E\left\{{\left|g_{m_{1}k}^{T}{\hat{g}}_{m_{1}k}^{*}+\alpha_{m}g_{m_{2}k}^{T}{\hat{g}}_{m_{2}k}^{*}\right|}^{2}\right.-\left.{\left|E\left\{g_{m_{1}k}^{T}{\hat{g}}_{m_{1}k}^{*}+\alpha_{m}g_{m_{2}k}^{T}{\hat{g}}_{m_{2}k}^{*}\right\}\right|}^{2}\right\}\\ =\left(N_{1}+\alpha_{m}^{2}N_{2}\right)\gamma_{mk}\beta_{mk}^{}+\left(N_{1}^{2}+\alpha_{m}^{2}N_{2}^{2}\right)\gamma_{mk}^{2}\\ -{\left(N_{1}^{}+\alpha_{m}N_{2}^{}\right)}^{2}\gamma_{mk}^{2}+2\alpha_{m}N_{1}N_{2}\gamma_{mk}^{2}\\ =\left(N_{1}+\alpha_{m}^{2}N_{2}\right)\gamma_{mk}\beta_{mk}^{}. \end{array}$$

Therefore, ${\mathrm{BU}}_{k}$ can be given as follows

(A6) $${\mathrm{BU}}_{k}=\rho_{d}\sum_{m=1}^{M}\eta_{mk}\left(N_{1}+\alpha_{m}^{2}N_{2}\right)\gamma_{mk}\beta_{mk}^{}.$$

(3) Compute ${\mathrm{UI}}_{kk^{\prime }}$: The term ${\mathrm{UI}}_{kk^{\prime }}$ can be rewritten as

(A7) $$\begin{array}{ll} UI_{kk^{\prime }} & =E\left\{{\left|\sqrt{\rho_{d}}\sum_{m=1}^{M}\sqrt{\eta_{mk^{\prime }}}g_{m_{1}k}^{T}{\hat{g}}_{m_{1}k^{\prime }}^{*}\right|}^{2}\right\}+E\left\{{\left|\sqrt{\rho_{d}}\sum_{m=1}^{M}\alpha_{m}\sqrt{\eta_{mk^{\prime }}}g_{m_{2}k}^{T}{\hat{g}}_{m_{2}k^{\prime }}^{*}\right|}^{2}\right\}\\ & =\rho_{d}E\left\{{\left|\sum_{m=1}^{M}\sqrt{\eta_{mk^{\prime }}}g_{m_{1}k}^{T}{\hat{g}}_{m_{1}k^{\prime }}^{*}\right|}^{2}+{\left|\sum_{m=1}^{M}\alpha_{m}\sqrt{\eta_{mk^{\prime }}}g_{m_{2}k}^{T}{\hat{g}}_{m_{2}k^{\prime }}^{*}\right|}^{2}\right\}\\ & +2\mathrm{Real}\left(\sum_{m=1}^{M}\sum_{n=1}^{M}\sqrt{\eta_{mk^{\prime }}\eta_{nk^{\prime }}}\alpha_{m}g_{m_{1}k}^{T}{\hat{g}}_{m_{1}k^{\prime }}^{*}g_{m_{2}k}^{T}{\hat{g}}_{m_{2}k^{\prime }}^{*}\right). \end{array}$$

To ${\mathrm{UI}}_{kk^{\prime }}$, we first need to calculate

(A8) $$\begin{array}{ll} E\left\{g_{m_{1}k}^{T}{\hat{g}}_{m_{1}k^{\prime }}^{*}\right\} & =E\left\{g_{m_{1}k}^{T}{\left(\sqrt{\tau \rho_{p}^{}}b_{mk^{\prime }}^{}\sum_{k^{\prime \prime }=1}^{K}g_{m_{1}k^{\prime \prime }}\varphi_{k}^{H}\varphi_{k^{\prime }}^{}+{\tilde{w}}_{m_{1}k^{\prime }}\right)}^{*}\right\}\\ & =\sqrt{\tau \rho_{p}^{}}b_{mk^{\prime }}^{}\sum_{k^{\prime \prime }=1}^{K}E\left(g_{m_{1}k}^{T}g{{}_{m_{1}k^{\prime }}^{*}}_{{}^{\prime }}\varphi_{k}^{T}\varphi_{k^{\prime }}^{*}\right)\\ & =N_{1}\sqrt{\tau \rho_{p}^{}}b_{mk^{\prime }}^{}\beta_{mk^{\prime }}^{}\frac{\beta_{mk}^{}}{\beta_{mk^{\prime }}^{}}\varphi_{k}^{T}\varphi_{k^{\prime }}^{*}=N_{1}\frac{\gamma_{mk^{\prime }}}{\beta_{mk^{\prime }}}\beta_{mk}^{}\varphi_{k}^{T}\varphi_{k^{\prime }}^{*}, \end{array}$$

where ${\tilde{w}}_{m_{1}k^{\prime }}$ includes *i.i.d.* CN(0,1) components [3]. With the above considerations, for the first term, we have

(A9) $$E\left\{{\left|\sum_{m=1}^{M}\sqrt{\eta_{mk^{\prime }}}g_{m_{1}k}^{T}{\hat{g}}_{m_{1}k^{\prime }}^{*}\right|}^{2}\right\}={\left|\varphi_{k}^{H}\varphi_{k^{\prime }}^{}\right|}^{2}\cdot N_{1}^{2}{\left(\sum_{m=1}^{M}\sqrt{\eta_{mk^{\prime }}^{}}\gamma_{mk^{\prime }}\frac{\beta_{mk}^{}}{\beta_{mk^{\prime }}^{}}\right)}^{2}+N_{1}^{}\sum_{m=1}^{M}\eta_{mk^{\prime }}^{}\gamma_{mk^{\prime }}\beta_{mk}^{}.$$

For the second term $E\left\{{\left|\sum_{m=1}^{M}\alpha_{m}\sqrt{\eta_{mk^{\prime }}}g_{m_{2}k}^{T}{\hat{g}}_{m_{2}k^{\prime }}^{*}\right|}^{2}\right\}$, using the same techniques as (A9), we have

(A10) $$\begin{array}{l} E\left\{{\left|\sum_{m=1}^{M}\alpha_{m}\sqrt{\eta_{mk^{\prime }}}g_{m_{2}k}^{T}{\hat{g}}_{m_{2}k^{\prime }}^{*}\right|}^{2}\right\}\\ ={\left|\varphi_{k}^{H}\varphi_{k^{\prime }}^{}\right|}^{2}\cdot N_{2}^{2}{\left(\sum_{m=1}^{M}\alpha_{m}\sqrt{\eta_{mk^{\prime }}^{}}\gamma_{mk^{\prime }}\frac{\beta_{mk}^{}}{\beta_{mk^{\prime }}^{}}\right)}^{2}+N_{2}^{}\sum_{m=1}^{M}\alpha_{m}^{2}\eta_{mk^{\prime }}^{}\gamma_{mk^{\prime }}\beta_{mk}^{}. \end{array}$$

For the third term, we have

(A11) $$\begin{array}{l} E\left\{\sum_{m=1}^{M}\sum_{n=1}^{M}\sqrt{\eta_{mk^{\prime }}\eta_{nk^{\prime }}}\alpha_{m}g_{m_{1}k}^{T}{\hat{g}}_{m_{1}k^{\prime }}^{*}\sqrt{\eta_{mk^{\prime }}}g_{m_{2}k}^{T}{\hat{g}}_{m_{2}k^{\prime }}^{*}\right\}\\ =\sum_{m=1}^{M}\sum_{n=1}^{M}\sqrt{\eta_{mk^{\prime }}\eta_{nk^{\prime }}}\alpha_{m}N_{1}^{}N_{2}^{}\left(\frac{\gamma_{mk^{\prime }}^{}}{\beta_{mk^{\prime }}^{}}\beta_{mk}^{}\right)\left(\frac{\gamma_{nk^{\prime }}^{}}{\beta_{nk^{\prime }}^{}}\beta_{nk}^{}\right){\left|\varphi_{k}^{H}\varphi_{k^{\prime }}^{}\right|}^{2}. \end{array}$$

After several algebraic manipulations, substituting (A9), (A10), and (A11) into (A7), the ${\mathrm{UI}}_{kk^{\prime }}$ can be achieved.

(4) Compute ${\mathrm{QI}}_{k}$: Since $E\left\{{\tilde{n}}_{m_{2}}{\tilde{n}}_{m_{2}}^{H}\right\}=\alpha_{m}\left(1-\alpha_{m}\right)\rho_{d}\sum_{k^{\prime }=1}^{K}\eta_{mk^{\prime }}\gamma_{mk^{\prime }}$, therefore, ${\mathrm{QI}}_{k}$ can be given as follows

(A12) $$\begin{array}{l} E\left\{{\left|\sum_{m=1}^{M}g_{m_{2}k}^{T}{\tilde{n}}_{m_{2}}\right|}^{2}\right\}=E\left\{\sum_{m=1}^{M}g_{m_{2}k}^{T}{\tilde{n}}_{m_{2}}{\tilde{n}}_{m_{2}}^{H}g_{m_{2}k}^{*}\right\}\\ =E\left\{\sum_{m=1}^{M}g_{m_{2}k}^{T}\left\{{\tilde{n}}_{m_{2}}{\tilde{n}}_{m_{2}}^{H}\right\}g_{m_{2}k}^{*}\right\}=N_{2}\rho_{d}\sum_{m=1}^{M}\alpha_{m}\left(1-\alpha_{m}\right)\beta_{mk}\sum_{k^{\prime }=1}^{K}\eta_{mk^{\prime }}\gamma_{mk^{\prime }}. \end{array}$$
